# Evaluation of patients’ satisfaction with food services and assessment of plate waste in Cypriot hospitals

**DOI:** 10.1017/jns.2025.10030

**Published:** 2025-08-15

**Authors:** Elena Hadjimbei, Stavrie Chrysostomou, Alexandros Heraclides, Konstantina Kouvari, Irene P. Tzanetakou

**Affiliations:** 1 Department of Life Sciences, School of Sciences, European University Cyprus, Nicosia, Cyprus; 2 Department of Health Sciences, School of Sciences, European University Cyprus, Nicosia, Cyprus

**Keywords:** Cyprus, Hospital food services, Hospital food waste, Patient satisfaction, Plate waste, ACHFPSQ, Acute Care Hospital Foodservice Patient Satisfaction Questionnaire, CNBC, Cyprus National Bioethics Committee, SHSO, Innovation Office of State Health Services Organisation, ORs, Odds Ratios, CI, Confidence Interval

## Abstract

Hospital food services and the resulting food waste impact patient satisfaction, health outcomes, healthcare costs, and the environment. This cross-sectional study assessed food waste and patient satisfaction in five public hospitals in Cyprus, involving 844 inpatients. Patient characteristics and responses to the 21-item Acute Care Hospital Foodservice Patient Satisfaction Questionnaire (ACHFPSQ) were recorded. Plate waste was evaluated using photographs and a five-point visual scale (0 to 1) to estimate food consumption. Hunger and overall satisfaction were also assessed. While 77.8% rated food services as good or very good, food quality received the most negative feedback. Only 31.2% finished their main dish entirely; 29.5% and 26.3% left ¼ and ½, respectively. For dessert, 48.2% finished it, while 13.3% left it untouched. These findings reveal a gap between general satisfaction and perceived food quality, underscoring the need for targeted public health strategies to enhance food quality and reduce waste in hospitals.

## Introduction

Hospital food services play a critical role in shaping the overall inpatient experience and supporting recovery through the provision of nutritionally adequate, safe, and palatable meals tailored to clinical needs^(1–3)^. Inadequate food intake in hospitals remains a global concern, with studies indicating that up to one-third of hospitalised patients are at risk of malnutrition^(4–6)^, contributing to poor outcomes such as delayed recovery, complications, longer hospital stays, and increased healthcare costs^(7,8)^.

Monitoring patient satisfaction with food services can serve as a key indicator for both service quality and nutritional risk, making it an essential element in healthcare quality improvement^(9,10)^. Factors influencing foodservice satisfaction include meal temperature, presentation, taste, portion size, menu variety, and responsiveness to patient preferences^(11–13)^. However, satisfaction levels vary widely across countries and institutions, with reported rates ranging from 30% to over 90%^(14–18)^. In Cyprus, data remain scarce, with limited evidence suggesting dissatisfaction in areas such as food variety and taste^(12)^.

Assessing inpatient satisfaction with food services is essential for identifying deficiencies and facilitating the continuous enhancement of hospital care, while simultaneously supporting optimal patient health outcomes. Nutritional management constitutes a fundamental component of the overall therapeutic regimen for numerous chronic and acute conditions. In certain instances, it may represent the primary therapeutic modality, as in the early stages of type 2 diabetes mellitus^(19)^. In other cases, the provision of specialised dietary interventions is critical to mitigating or preventing adverse effects associated with treatment, such as in oncology patients receiving chemotherapy^(20)^. Accordingly, the delivery of appropriate meals should be regarded as an integral aspect of inpatient medical care, with the capacity to promote and support patient recovery^(21,22)^.

Importantly, hospital foodservice must also balance patient satisfaction with nutritional adequacy and environmental sustainability. Despite high satisfaction scores, food waste remains a persistent issue in healthcare settings, with up to 50% of food being discarded^(23,24)^. Reasons include reduced appetite, illness, poor food quality, and meal timing^(25)^, highlighting the need for targeted interventions. Reducing hospital food waste offers benefits beyond nutrition, including cost savings and reduced environmental impact^(26–28)^. Nevertheless, standardised strategies are lacking, and many systems are driven primarily by economic considerations^(29,30)^.

Given the limited national data and growing emphasis on food quality and waste reduction in healthcare, the present study aimed to evaluate plate waste and patient satisfaction with food services in public hospitals in Cyprus, contributing evidence for future public health and hospital management strategies.

## Materials and methods

### Design and participants

A cross-sectional study was carried out in 5 public hospitals in Cyprus (Larnaca General Hospital, Nicosia General Hospital, Paralimni General Hospital, Limassol General Hospital, Paphos General Hospital) between July 2022 and March 2023. **This study was conducted according to the guidelines laid down in the Declaration of Helsinki, and all procedures involving research study participants were approved by the** Cyprus National Bioethics Committee (CNBC) (EEBK EΠ 2021.01.255). The study was also approved by the Research and Innovation Office of State Health Services Organisation (SHSO) and by all the executive general managers of the participating hospitals.

Participation was anonymous, and all participants were informed about the aim and objectives of the study before accepting to taking part. Anonymity in research data management, was ensured through the technique of data masking by eliminating names, addresses, phone numbers, email addresses, and other information that could directly link data to an individual, providing a sole 4-digit code for each patient. Additionally, strong access controls and secure data storage, were crucial for maintaining participant confidentiality and privacy. Participants gave their informed consent for being involved in the study, by signing an informed consent form. Only hospitalised patients aged > 18 years old with a minimum stay of 2 days, were eligible for inclusion in the current study. Moreover, patients who were not fed orally, were excluded along with patients on dietary restrictions or prescriptions.

Public hospitals in Cyprus operate under a common in-house food service model, whereby each hospital manages its own food preparation and distribution internally^(31)^. This shared structure ensures a consistent framework across hospitals, although some variations in menu planning and daily operations exist due to local autonomy. As such, while the core system is aligned, complete comparability between hospitals may be limited^(32)^.

Data were collected using the Acute Care Hospital Foodservice Patient Satisfaction Questionnaire (ACHFPSQ). The ACHFPSQ is a reliable validated tool for measuring patient satisfaction with food services^(33)^. The questionnaire includes, general information about the patients such as, gender, age, height, weight, educational attainment. Weight and height were self-reported by the participants, a method widely accepted in epidemiological research despite potential reporting bias^(34,35)^. Patient satisfaction was evaluated using a 5-point Likert scale with the options ‘Always’, ‘Often’, ‘Sometimes’, ‘Rarely’ and ‘Never’. The patient was asked to rate statements related to 4 key dimensions of feeding services: i) Food quality (taste, texture, appearance, how vegetables and meat are cooked), ii) Staff/service issues (politeness, cleanliness, willingness to help), iii) Meal service quality (cleanliness and quality of the tray, temperature of cold and hot food and drinks), iv) The physical environment (smells and noise in the hospital, interruptions during mealtime). The amount of food and the feeling of hunger were also assessed through a specific set of questions (i.e. A) I receive enough food, B) I still feel hungry after my meal, C) I feel hungry in between meals), while at the end there was a question regarding overall satisfaction with the food service.

### Visual estimation method of plate waste

The assessment of plate waste, was performed by observing and taking photographs of the amount of food of the main dish and the dessert remaining on the patients’ plates, after lunch, compared with the original food plate.

A meal intake observation tool using a five-point visual scale (0, ¼, ½, ¾, 1) was used, to record the volume of each meal consumed by the patient. Code of zero indicated that the patient eats all the food on the plate, a code of 1/4 indicated that the patient leaves 1/4 of the food on the plate, a code of 1/2 indicated that the patient leaves 1/2 of the food on the plate, a code of 3/4 indicated that the patient leaves 3/4 of the food on the plate, and a code of 1 indicated that the patient leaves all the food on the plate. The validity and reliability of the observation tool have been established in previous studies^(36)^. In addition, inter-rater reliability was evaluated in the present study and demonstrated substantial agreement, with a weighted Cohen’s kappa of 0.72 (95% CI: 0.65–0.79).

All data were collected by the University’s Nutrition and Dietetics students, and data collection was supervised by the hospital dietitian. Students were provided with 1 day of training, regarding the data collection methodology.

### Statistical analysis

Main characteristics of the 844 patients included in the study, as well as their responses on the 22 ACHFPSQ items (21 scale items plus 1 overall satisfaction question) are presented using proportions and absolute numbers.

Exploratory factor analysis was performed on the 21 ACHFPSQ scale items. Initially, an intercorrelation matrix was constructed, and the Bartlett’s test of sphericity was used to evaluate factorability in the intercorrelation matrix. Factor analysis was performed setting the minimum number of factors to 5, based on a varimax rotation in order to maximise items in factor patterns. A cut-off of 0.3 on the factor loadings of items was used as a criterion of inclusion in the relevant factor. For items that loaded in more than one factor, the higher loading score was considered, and the item was included in the relevant factor. Factors were only considered if they contained a minimum of 3 items.

Following the ACHFPSQ guidelines, a factor score was calculated only among participants who had valid answers in all relevant items. Eigenvalues were estimated, representing the common variance of the observed items each factor explains. The explained variance for each factor, representing the proportion of individual differences accounted for by the common factors, was also estimated.

Summary statistics for the 5 derived ACHFPSQ dimensions (factors) in the study sample were estimated using measures of central tendency (mean and median) and measures of dispersion (standard deviation, interquartile range), while the distribution is presented using histograms.

For investigating the association between patient characteristics and the 5 ACHFPSQ dimensions multiple linear regression was used. In this analysis, patient characteristics were included as categorical independent variables and the 5 ACHFPSQ dimensions as numeric dependent variables, in separate models, adjusting for age, gender, district, and educational attainment. Results from this analysis represent mean differences (positive values indicate that the comparison category has higher satisfaction than the reference category, while negative values indicate that the comparison category has lower satisfaction than the reference category).

The association between patient characteristics and the single question evaluating the overall satisfaction with hospital foodservice (ordinal variable: very poor, poor, okay, good, very good), a contingency table was constructed, and a Pearson’s chi-squared test was used to derive a p-value for evaluating statistical significance of associations.

To investigate the association between the 5 ACHFPSQ dimensions and main dish leftover (i.e. whether patients left more than half of their food on the plate during their hospital stay), multiple logistic regression was used. In this analysis, the 5 ACHFPSQ dimensions were included as numeric independent variables, and main dish leftover was included as binary dependent variable, in separate models, adjusting for age, gender, district, and educational attainment. Results from this analysis are presented as Odds Ratios (ORs above 1 indicate that the higher the score the higher the likelihood of leaving more than half of food in the plate; ORs below 1 indicate that the higher the score the lower the likelihood of leaving more than half of food in the plate).

Statistical significance was evaluated using the p-value at a 5% significance level, as well as the 95% Confidence Interval (CI). All statistical analyses were performed using the R statistical software environment, version 4.3.0.

## Results

### Participant characteristics

Of the 844 patients surveyed, the majority were men (57%) and over the age of 70 (38%). Most were married (71%) and retired (49%), with 45% classified as overweight and 20% as obese. Educational attainment varied, with 31% having completed only primary education, and income levels clustered primarily in the 501–1000 euro/month range. Full demographic details are provided in Table 1.

**Table 1.** Characteristics of the 844 patients included in the study

Participant characteristic	% (n)
District	
Nicosia	5.8% (49)
Larnaca	33.9% (286)
Limassol	20.5% (173)
Paphos	22.4% (189)
Famagusta	17.4% (147)
Age group	
18–30	9.4% (79)
31–50	17.1% (144)
51–70	35.2% (297)
> 70	38.3% (324)
Gender	
Men	57.1% (482)
Women	42.9% (362)
Marital status	
Married/Co-habiting	70.6% (594)
Single	11.9% (100)
Divorced/Separated	6.3% (53)
Widowed	11.2% (94)
Educational attainment	
Primary school	30.9% (261)
Secondary school	17.6% (149)
High school	26.1% (220)
College	10.0% (84)
University – Undergraduate	13.4% (113)
University – Postgraduate	2.0% (17)
Occupation	
Private employee	25.1% (211)
Public employee	7.8% (66)
Freelance	6.4% (54)
Unemployed/Housewife	9.9% (84)
Student/Other	2.4% (20)
Retired	48.5% (409)
Health professional	
Yes	3.8% (32)
No	96.2% (811)
Income (Euros)	
No salary	12.1% (101)
< 500	16.7% (140)
501–1000	35.2% (295)
1001–1500	22.3% (187)
> 1500	13.7% (115)
Religion	
Orthodox Christian	89.3% (754)
Catholic Christian	4.7% (40)
Muslim	3.3% (28)
Other	2.7% (22)
Body weight	
Underweight	1.4% (11)
Normal weight	33.1% (278)
Overweight	45.5% (382)
Obesity	20.0% (168)
Leftovers (main dish)	
None	31.2% (262)
1/4	29.5% (248)
1/2	26.3% (221)
3/4	10.0% (84)
All	3.1% (26)
Leftovers (desert)	
None	48.2% (405)
1/4	16.7% (140)
1/2	14.4% (121)
3/4	7.4% (62)
All	13.3% (112)

### Patient satisfaction with food services

The last question in Table 2 presents the overall patient satisfaction with the food service, which in the literature^(37,38)^ has been assessed on its own (i.e. did not form part of a scale). Overall satisfaction with hospital food services was rated as ‘very good’ or ‘good’ by 78% of respondents. Satisfaction was highest for staff/service interactions and meal temperature, with over 90% rating these dimensions positively. However, aspects of food quality—including variety and the ability to choose healthy meals—were rated less favourably (see Table 2). Notably, 69% of patients reported limited ability to choose healthy meals, and 42% found the meat often tough or dry.

**Table 2.** Percentage distributions of the ACHFPSQ items answered in the study sample

ACHFPSQ items	% (n)				
	Always	Often	Sometimes	Rarely	Never
The hospital food has been as good as I expected	41.0% (346)	25.7% (217)	21.4% (180)	6.4% (54)	5.5% (46)
The crockery and cutlery are chipped and/or stained	12.0% (101)	0.8% (7)	1.3% (11)	4.4% (37)	81.5% (688)
The staff who deliver my meals are neat and clean	93.1% (786)	5.0% (42)	1.3% (11)	0.4% (3)	0.2% (2)
The hospital smells stop me from enjoying my meals	1.3% (11)	2.0% (17)	9.7% (82)	11.3% (95)	75.7% (639)
I am able to choose a healthy meal in hospital	19.3% (162)	11.7% (98)	8.5% (71)	5.5% (46)	55.1% (462)
I am disturbed by the noise of finished meal trays being removed	0.6% (5)	0.8% (7)	6.8% (57)	11.3% (95)	80.6% (680)
The cold drinks are just the right temperature	60.8% (510)	11.1% (93)	4.6% (39)	0.8% (7)	22.6% (190)
I like the way the vegetables are cooked	37.1% (307)	22.0% (182)	21.1% (175)	7.7% (64)	12.1% (100)
The meals taste nice	38.9% (328)	24.0% (202)	25.4% (214)	8.3% (70)	3.4% (29)
The hot drinks are just the right temperature	76.1% (641)	14.7% (124)	6.5% (55)	1.2% (10)	1.4% (12)
The staff who take away my finished meal tray are friendly and polite	93.0% (784)	4.2% (35)	2.4% (20)	0.1% (1)	0.4% (3)
I like to be able to choose different sized meals	20.9% (176)	7.5% (63)	12.1% (102)	12.1% (102)	47.4% (400)
The menu has enough variety for me to choose meals that I want to eat	20.7% (174)	14.3% (120)	14.3% (120)	8.2% (69)	42.4% (356)
The cold foods are the right temperature	80.5% (676)	11.9% (100)	4.9% (41)	0.2% (2)	2.5% (21)
The staff who deliver menus are helpful	94.4% (796)	3.2% (27)	1.5% (13)	0.4% (3)	0.5% (4)
The meals have excellent and distinct flavours	45.7% (385)	21.0% (177)	25.1% (212)	5.3% (45)	2.8% (24)
The hot foods are just the right temperature	73.6% (620)	16.6% (140)	7.0% (59)	1.0% (8)	1.8% (15)
The meat is tough and dry	7.5% (63)	12.0% (101)	23.1% (194)	15.4% (129)	42.0% (352)
I received enough food	75.9% (640)	15.5% (131)	5.5% (46)	1.7% (14)	1.4% (12)
I am still hungry after finishing the meal	2.8% (24)	3.6% (30)	5.2% (44)	11.4% (96)	77.0% (649)
I am hungry between consecutive meals	3.3% (28)	4.4% (37)	9.5% (80)	17.0% (143)	65.8% (555)
	Very good	Good	Okay	Poor	Very Poor
Overall, how would you rate your satisfaction with the food service	40.6% (341)	37.2% (312)	18.7% (157)	1.9% (16)	1.5% (13)

### Factor analysis of satisfaction dimensions

Exploratory factor analysis identified five distinct dimensions of foodservice satisfaction: Food Quality, Meal Service Quality, Staff/Service Issues, Hunger and Quantity, and Physical Environment, consistent with previous applications of ACHFPSQ in other populations^(33,38)^. These five factors explained 42% of the total variance in satisfaction scores (Table 3).

**Table 3.** Factor loadings of the ACHFPSQ items obtained by factor analysis in the study sample

ACHFPSQ items	Factors				
	Food quality	Meal service quality	Staff/service issues	Hunger and Quantity	Physical environment
The hospital food has been as good as I expected	0.64				
The crockery and cutlery are chipped and/or stained					0.44
The staff who deliver my meals are neat and clean			0.62		
The hospital smells stop me from enjoying my meals					0.32
I am able to choose a healthy meal in hospital	0.48				
I am disturbed by the noise of finished meal trays being removed					0.35
The cold drinks are just the right temperature		0.49			
I like the way the vegetables are cooked	0.38				
The meals taste nice	0.72				
The hot drinks are just the right temperature		0.68			
The staff who take away my finished meal tray are friendly and polite			0.80		
I like to be able to choose different sized meals	–	–	–	–	–
The menu has enough variety for me to choose meals that I want to eat	0.51				
The cold foods are the right temperature		0.73			
The staff who deliver menus are helpful			0.59		
The meals have excellent and distinct flavours	0.76				
The hot foods are just the right temperature		0.72			
The meat is tough and dry	0.33				
I received enough food				0.43	
I am still hungry after finishing the meal				0.89	
I am hungry between consecutive meals				0.64	
Eigenvalues	2.72	2.06	1.56	1.70	0.94
Explained variance	13%	10%	7%	8%	4%

### Distribution of satisfaction scores

With the exception of Food Quality, which followed a normal distribution, most satisfaction dimensions were skewed toward high scores. Median scores for Staff/Service Issues, Meal Service Quality, Hunger and Quantity, and Physical Environment reached their maximum possible values, indicating overall high satisfaction (Table 4, Supplementary Figure 1).

**Table 4.** Summary statistics for the 5 Acute Care Hospital Foodservice Patient Satisfaction dimensions in the study sample

ACHFPSQ factor^*^	Min	Max	Mean	Median	S.dev	IQR
Food quality	7.0	35.0	24.1	24.0	6.0	10
Meal service quality	4.0	20.0	17.8	20.0	3.1	4
Staff/service issues	3.0	15.0	14.7	15.0	1.0	0
Hunger and quantity	3.0	15.0	13.6	15.0	2.3	2
Physical environment	3.0	15.0	13.7	15.0	2.0	2
Overall satisfaction	1.0	5.0	4.1	4	0.9	1

* For all ACHFPSQ factors (dimensions), the higher the score the higher the satisfaction of the patients.

### Participant characteristics in relation to foodservice satisfaction

Satisfaction varied significantly across districts, with patients from the capital city of Nicosia, showing the lowest foodservice satisfaction and patients from Limassol and even more so Paphos, consistently reporting higher satisfaction across all dimensions. No clear trends in satisfaction were observed with age, apart from meal service quality satisfaction which appeared lower among middle-aged to older compared to younger patients. Women were less satisfied with food quality and the physical environment but reported higher satisfaction regarding hunger and food quantity. Divorced or separated patients reported lower food quality, as well as hunger and food quantity satisfaction. Higher educational attainment was associated with lower satisfaction in food quality. Obese and overweight patients tended to rate food quality more positively and food quantity and satiety less positively than those with normal body weight. Other patient characteristics, such as occupation, income, and religion did not show clear patterns as regards foodservice satisfaction (Table 5).

**Table 5.** Linear regression results showing adjusted mean difference (95% confidence interval) for the association between patient characteristics and the Acute Care Hospital Foodservice Patient Satisfaction dimensions

	Mean difference (95% CI)^*^				
Participant characteristic	Food quality	Meal service quality	Staff/service issues	Hunger and quantity	Physical environment
District					
Nicosia	Ref	Ref	Ref	Ref	Ref
Larnaca	–1.14 (–2.70, 0.43)	1.35 (0.54, 2.16)	0.35 (0.04, 0.66)	0.15 (–0.51, 0.82)	1.08 (0.52, 1.64)
Limassol	2.69 (1.06, 4.31)	3.32 (2.47, 4.16)	0.33 (0.00, 0.65)	0.91 (0.21, 1.61)	1.26 (0.67, 1.84)
Paphos	6.80 (5.19, 8.41)	4.92 (4.09, 5.76)	0.52 (0.19, 0.84)	1.80 (1.11, 2.49)	1.28 (0.70, 1.86)
Famagusta	–0.39 (–2.05, 1.27)	4.56 (3.70, 5.42)	0.62 (0.29, 0.95)	0.36 (–0.35, 1.07)	–0.79 (–1.39, –0.19)
Age group					
18–30	Ref	Ref	Ref	Ref	Ref
31–50	–1.38 (–2.80, 0.04)	–0.95 (–1.69, –0.20)	0.05 (–0.23, 0.33)	–0.07 (–0.68, 0.54)	–0.20 (–0.71, 0.31)
51–70	–0.31 (–1.58, 0.97)	–0.70 (–1.37, –0.03)	0.02 (–0.24, 0.28)	–0.04 (–0.59, 0.51)	–0.05 (–0.51, 0.41)
> 70	0.84 (–0.42, 2.10)	–0.45 (–1.11, 0.21)	0.19 (–0.07, 0.44)	0.61 (0.07, 1.15)	0.26 (–0.20, 0.71)
Gender					
Men	Ref	Ref	Ref	Ref	Ref
Women	–0.71 (–1.42, –0.01)	–0.13 (–0.50, 0.24)	–0.10 (–0.24, 0.04)	0.54 (0.24, 0.84)	–0.34 (–0.59, –0.08)
Marital status					
Married/Co-habiting	Ref	Ref	Ref	Ref	Ref
Single	0.23 (–1.00, 1.46)	0.31 (–0.33, 0.95)	0.21 (–0.03, 0.45)	–0.06 (–0.58, 0.47)	0.34 (–0.10, 0.78)
Divorced/Separated	–1.92 (–3.38, –0.46)	0.15 (–0.60, 0.90)	–0.02 (–0.30, 0.26)	–0.92 (–1.54, –0.30)	–0.29 (–0.80, 0.23)
Widowed	0.56 (–0.66, 1.77)	0.07 (–0.56, 0.69)	0.16 (–0.08, 0.39)	0.29 (–0.23, 0.81)	0.39 (–0.04, 0.82)
Educational attainment					
Primary school	Ref	Ref	Ref	Ref	Ref
Secondary school	–1.03 (–2.08, 0.02)	–0.52 (–1.06, 0.03)	–0.14 (–0.35, 0.07)	–0.52 (–0.97, –0.07)	–0.26 (–0.64, 0.11)
High school	–1.32 (–2.34, –0.29)	–0.25 (–0.78, 0.28)	–0.02 (–0.22, 0.18)	–0.20 (–0.64, 0.24)	–0.13 (–0.49, 0.24)
College	–1.87 (–3.21, –0.52)	–0.30 (–1.00, 0.41)	0.15 (–0.12, 0.42)	–0.42 (–1.00, 0.16)	–0.03 (–0.52, 0.45)
University	–1.77 (–3.02, –0.52)	–0.44 (–1.10, 0.21)	0.06 (–0.19, 0.31)	–0.40 (–0.94, 0.14)	0.14 (–0.32, 0.59)
Occupation					
Private employee	Ref	Ref	Ref	Ref	Ref
Public employee	0.06 (–1.39, 1.51)	–0.29 (–1.04, 0.46)	–0.20 (–0.49, 0.09)	0.40 (–0.22, 1.02)	0.32 (–0.19, 0.84)
Freelance	–0.37 (–1.92, 1.17)	–0.51 (–1.33, 0.30)	–0.06 (–0.37, 0.24)	0.07 (–0.59, 0.73)	0.16 (–0.39, 0.71)
Unemployed/Housewife	–0.76 (–2.12, 0.59)	–0.40 (–1.10, 0.29)	–0.23 (–0.50, 0.04)	–0.14 (–0.72, 0.43)	–0.13 (–0.62, 0.35)
Student/Other	–0.55 (–3.05, 1.96)	0.20 (–1.11, 1.50)	–0.01 (–0.51, 0.50)	0.54 (–0.54, 1.62)	0.45 (–0.46, 1.35)
Retired	0.43 (–0.82, 1.67)	0.32 (–0.32, 0.96)	–0.13 (–0.37, 0.12)	0.14 (–0.39, 0.67)	0.49 (0.05, 0.93)
Health professional					
Yes	Ref	Ref	Ref	Ref	Ref
No	0.35 (–1.47, 2.17)	–0.26 (–1.23, 0.71)	0.17 (–0.20, 0.53)	–0.64 (–1.43, 0.15)	–0.05 (–0.71, 0.61)
Income (Euros)					
No salary	Ref	Ref	Ref	Ref	Ref
< 500	1.02 (–0.49, 2.53)	0.51 (–0.27, 1.29)	0.15 (–0.15, 0.45)	0.48 (–0.16, 1.12)	0.13 (–0.42, 0.67)
501–1000	0.49 (–0.80, 1.79)	0.19 (–0.48, 0.86)	0.13 (–0.13, 0.39)	0.38 (–0.17, 0.93)	0.16 (–0.30, 0.63)
1001–1500	1.07 (–0.21, 2.36)	0.15 (–0.51, 0.82)	0.18 (–0.08, 0.43)	0.28 (–0.27, 0.83)	0.10 (–0.36, 0.57)
> 1500	–0.99 (–2.42, 0.44)	–0.45 (–1.19, 0.29)	0.15 (–0.14, 0.43)	0.16 (–0.44, 0.77)	0.51 (–0.00, 1.03)
Religion					
Orthodox Christian	Ref	Ref	Ref	Ref	Ref
Catholic Christian	–1.02 (–2.67, 0.63)	0.02 (–0.86, 0.89)	–0.72 (–1.05, –0.39)	–0.89 (–1.60, –0.18)	0.06 (–0.54, 0.66)
Muslim	–0.29 (–2.33, 1.75)	–0.06 (–1.11, 0.99)	–0.57 (–0.97, –0.17)	0.00 (–0.87, 0.86)	–0.72 (–1.44, 0.01)
Other	1.46 (–0.76, 3.68)	0.30 (–0.87, 1.46)	0.09 (–0.36, 0.53)	0.33 (–0.63, 1.28)	0.18 (–0.63, 0.99)
Body weight					
Normal weight^**^	Ref	Ref	Ref	Ref	Ref
Overweight	1.33 (0.53, 2.13)	0.19 (–0.23, 0.61)	0.11 (–0.05, 0.27)	–0.14 (–0.48, 0.21)	0.24 (–0.05, 0.53)
Obesity	1.93 (0.92, 2.94)	0.11 (–0.41, 0.64)	0.06 (–0.14, 0.27)	–0.47 (–0.90, –0.04)	0.36 (–0.00, 0.72)

* Mean difference (95% CI) estimated using multiple linear regression treating all ACHFPSQ factors (dimensions), in turn, as numeric dependent variables and the different patient characteristics as categorical independent variables, adjusting for age, gender, district, and educational attainment.

** The category ‘Normal weight’ includes a small number of underweight (BMI < 18.5 kg/m^2^) individuals.

### Food waste (plate waste) & associations with foodservice satisfaction

Only 31% of patients finished their main dish, while the rest left between ¼ and all of the food uneaten. Similar patterns were seen for desserts, with 52% of patients leaving at least some food on the plate (Table 1).

Higher satisfaction scores in Food Quality and Hunger/Quantity were significantly associated with reduced likelihood of main meal food waste (leaving more than half the main dish uneaten), as was the overall food satisfaction scale. A one-point increase in overall satisfaction was linked to a 45% decrease in the odds of high food waste, highlighting the role of perceived food quality in meal consumption. Satisfaction with the physical environment also showed a negative trend in its association with food waste, with the association not reaching statistical significance (Table 6).

**Table 6.** Adjusted odds ratios (95% confidence interval) for the association between the Acute Care Hospital Foodservice Patient Satisfaction dimensions and main dish leftover

ACHFPSQ factor	Odds ratio (95% CI)^*^
Food quality	0.92 (0.88, 0.95)
Meal service quality	0.98 (0.91, 1.05)
Hunger and quantity	0.84 (0.78, 0.91)
Staff/service issues	1.01 (0.84, 1.27)
Physical environment	0.92 (0.84, 1.02)
Overall satisfaction	0.55 (0.44, 0.69)

* Odds Ratios (95% CI) estimated using multiple logistic regression treating more than half of portion left in plate as a binary dependent variable and all ACHFPSQ factors (dimensions), in turn, as numeric independent variables, adjusting for age, gender, district, and educational attainment.

## Discussion

This study investigated patient satisfaction with hospital food services and quantified plate waste across five public hospitals in Cyprus. Overall satisfaction was relatively high (77.8% rated services as ‘good’ or ‘very good’), yet food waste remained significant, with 68.8% of patients leaving some portion of their main dish uneaten. This duality underscores the complexity of foodservice performance, where satisfaction metrics do not always translate to adequate intake or efficient resource use.

The study’s results are consistent with international findings showing similarly high satisfaction ratings in countries such as Sweden^(18)^ and Saudi Arabia^(17)^, but also confirm prior observations that food quality remains a weaker aspect of satisfaction^(17,18,22,37–39)^. This dimension—capturing taste, variety, and texture—showed the most variability in patient responses, in line with findings from Trinca et al. (2022)^(40)^, who identified sensory food attributes and mealtime experience as core drivers of satisfaction. These nuanced perceptions may explain the observed gap between reported satisfaction and actual consumption.

Furthermore, the majority of the study participants (45.5%) were overweight, and 20% obese. Our results are in line with previous studies which also indicated high levels of overweight and obese patients^(41,42)^. Previous research indicates that malnutrition or the risk of malnutrition may be readily missed, particularly in patients who are overweight or obese, and that malnutrition associated with disease status may be a predictor of worse patient outcomes regardless of body mass index^(42,43)^.

Our multivariate analysis further revealed important socio-demographic patterns. Geographic location emerged as a significant determinant, with participants from Paphos reporting higher satisfaction across all domains. This may reflect regional differences in hospital catering services, cultural alignment with menu offerings, or resource allocation—patterns also observed in hospital studies in Italy^(38)^ and Canada^(40)^. Gender differences were also evident; women reported lower satisfaction with food quality and physical environment, but higher satisfaction in hunger and quantity. This aligns with prior research suggesting women tend to be more critical of food sensory aspects and presentation^(44)^, while also exhibiting different appetite patterns or caloric needs. Previous published work assessing socio-demographic traits associated with satisfaction of hospital meal service, has shown conflicting results, with certain studies not revealing discernible trends^(37,38,44,45)^, while others show either minor or major differences between groups of patients in terms of sex, age, gender, education, length of stay, appetite, perception of degree of control over health, and belief that food influences one’s health status and level of food intake were detected^(18,46)^. The complexity and diversity of resident food and taste preferences is indeed affected by many individual characteristics ranging from demographic to more complex genetic differences^(47)^.

Importantly, educational attainment was inversely related to food quality satisfaction, a trend previously reported by Naithani et al. (2009)^(25)^ and Capra et al. (2005)^(33)^. It is likely that more educated patients have higher expectations for food variety, nutritional content, and customisation. Conversely, overweight and obese participants reported higher satisfaction with food quality, potentially due to different taste preferences, portion size adequacy, or lower comparative standards. However, obese patients reported lower satisfaction with hunger/quantity, suggesting potential mismatches between standard hospital portions and perceived needs.

The relationship between satisfaction and plate waste provides critical insight into service inefficiencies. Consistent with international studies^(48,49)^, we found that food quality and perceived adequacy were strongly associated with lower food waste^(50–52)^. The better the perception of food quality, the more likely the food was consumed. However, our study also highlights that satisfaction with service attributes—such as staff politeness or cleanliness—can inflate overall satisfaction scores despite low food intake. This phenomenon, also observed in studies from Sweden and Iran^(18,48)^, suggests that subjective satisfaction and objective consumption must be interpreted together to evaluate hospital foodservice effectiveness accurately.

From a public health perspective, improving hospital food quality can yield multi-level benefits: enhancing patient intake, reducing malnutrition risk, and decreasing waste. A dual approach is warranted—one that integrates patient feedback and aligns meal services with clinical nutrition standards^(19)^. Studies by Doorduijn et al. (2016)^(21)^ and MacKenzie-Shalders et al. (2020)^(53)^ demonstrate that flexible systems like room service or bedside ordering improve intake and satisfaction without compromising quality^(54,55)^. These models offer promising directions for Cyprus, where centralised, inflexible catering practices currently dominate.

The environmental implications of food waste must also be emphasised. Uneaten hospital food contributes significantly to greenhouse gas emissions through production, transport, and methane release from organic waste^(26,56)^. Healthcare institutions—especially those with large-scale meal operations—must view food waste not only as a financial burden but also as a sustainability issue aligned with broader global targets, including the UN Sustainable Development Goals^(57)^. Reducing food waste through quality improvement and patient-centred services is therefore both a health and environmental priority.

Limitations of this study include its cross-sectional design, which restricts causal inference, and reliance on self-reported data, which may introduce recall or social desirability bias^(58)^. Additionally, convenience sampling, while logistically practical, may affect generalizability. However, this study is the first in Cyprus to combine quantitative assessment of satisfaction with visual estimation of plate waste, and provides an important baseline for future national monitoring.

In conclusion, our findings reinforce the need for a comprehensive and tailored foodservice model that emphasises quality, patient choice, and sustainability. Further research should explore longitudinal outcomes of foodservice interventions and leverage validated tools—such as those proposed by Trinca et al. (2022)^(40)^—to standardise satisfaction and intake assessments across healthcare settings.

### Conclusion

This study demonstrates that while overall patient satisfaction with hospital food services in Cyprus is high, significant dissatisfaction persists regarding food quality, and substantial plate waste remains a pervasive issue. The observed disconnect between reported satisfaction and actual consumption underscores the complexity of evaluating foodservice effectiveness. Addressing these gaps will require coordinated efforts to improve menu variety, sensory quality, and nutritional value, while also implementing patient-centred approaches that can reduce waste and promote adequate intake. Tailored public health strategies and more flexible foodservice models should be prioritised to enhance patient outcomes and advance sustainability objectives within the healthcare sector.

## Supporting information

Hadjimbei et al. supplementary material Hadjimbei et al. supplementary material

## Supplementary material

To view supplementary material for this article, please visit https://doi.org/10.1017/jns.2025.10030
