# Supplementary material for: Evaluation of patients’ satisfaction with food services and assessment of plate waste in Cypriot hospitals
Source: J Nutr Sci. 2025 Aug 15;14:e57. doi: 10.1017/jns.2025.10030 (PMC12361678; doi:10.1017/jns.2025.10030)
Supplement: Hadjimbei et al. supplementary material [file S204867902510030Xsup001.docx]

**Supplementary Figure 1.** **Distribution of the 5 Acute Care Hospital Foodservice Patient Satisfaction dimensions in the study sample**
